# Building Capacity for Inclusive Dementia Care: The development of the Alzheimer's Society of Ontario Health Equity Framework for Vulnerable Seniors Living with Dementia

**DOI:** 10.1002/alz70858_107669

**Published:** 2025-12-26

**Authors:** Ngozi Faith Iroanyah

**Affiliations:** ^1^ Alzheimer Society of Ontario, Toronto, ON, Canada

## Abstract

**Background:**

As the most populous province in the country, Ontario is home to over 16,000,000, with almost 3.0 million people aged 65+. Ontario is also home to a rich diverse population, including the most amount of immigrant communities, and largest population of Indigenous people.

Aging diverse populations have unique age‐related health challenges and needs that require targeted responses. Ontario accounts for over 300,000 of the approximately 700,000 dementia cases.

To support priority populations, the Alzheimer Society of Ontario has developed the Health Equity Framework for Vulnerable Seniors Living with Dementia to support the 26 local provincial Alzheimer societies in the development and implementation of health equity initiatives.

**Methods:**

The health equity framework was developed in 3 phases. Phase 1 was a review of theoretical equity‐based frameworks that focused on themes of social justice, anti‐oppression, and social determinants of health to support vulnerable populations. Frameworks that addressed seniors’ health, and dementia care were also reviewed. This included frameworks that considered person‐centered approaches, the 6 domains of health and wellness, assets and strength bases approaches. There was no model found that discussed dementia and equity specifically. Phase 2 consists of in‐person and virtual public consultations with local Alzheimer Society senior leadership and staff over a 6‐month period, and a qualitative data analysis of the findings. Additional consultations with industry experts are also part of phase 2 to gain industry perspectives on health equity and dementia. Phase 3 is the implementation of the framework, and evaluation of federation uptake and efficacy. This will be conducted in the spring of 2025.

**Results:**

At the time of this abstract submission, 23 out of 26 societies were consulted, with further consultations to happen in the winter of 2024. Industry expert consultations will also be consulted in the spring of 2025. Initial review of the consultations revealed broader themes around ways to conduct community engagement, organizational gaps, and inclusive program development.

**Conclusion:**

Findings from the consultations will be used to develop the framework to increase organizational capacity and competency, and systems level responsiveness to health equity gaps.

